# Navigating abortion law dilemmas: experiences and attitudes among Ethiopian health care professionals

**DOI:** 10.1186/s12910-021-00735-y

**Published:** 2021-12-18

**Authors:** Demelash Bezabih Ewnetu, Viva Combs Thorsen, Jan Helge Solbakk, Morten Magelssen

**Affiliations:** 1 Department of Physiology, St Paul’s Hospital Millennium Medical College, Addis Ababa, Ethiopia; 2 Centre for Medical Ethics, Institute of Health and Society, University of Oslo, Oslo, Norway; 3 Department of Community Medicine and Global Health, Institute of Health and Society, University of Oslo, Oslo, Norway

**Keywords:** Abortion, Abortion law, Ethical/moral challenges, Ethical/moral dilemmas, Ethiopia, Fetal abnormalities

## Abstract

**Background:**

Ethiopia’s 2005 abortion law improved access to legal abortion. In this study we examine the experiences of abortion providers with the revised abortion law, including how they view and resolve perceived moral challenges.

**Methods:**

Thirty healthcare professionals involved in abortion provisions in Addis Ababa were interviewed. Transcripts were analyzed using systematic text condensation, a qualitative analysis framework.

**Results:**

Most participants considered the 2005 abortion law a clear improvement—yet it does not solve all problems and has led to new dilemmas. As a main finding, the law appears to have opened a large space for professionals’ individual interpretation and discretion concerning whether criteria for abortion are met or not. Regarding abortion for fetal abnormalities, participants support the woman’s authority in deciding whether to choose abortion or not, although several saw these decisions as moral dilemmas. All thought that abortion was a justified choice when a diagnosis of fetal abnormality had been made.

**Conclusion:**

Ethiopian practitioners experience moral dilemmas in connection with abortion. The law places significant authority, burden and responsibility on each practitioner.

## Background

### Abortion in Ethiopia

The 2003 Protocol to the African Charter on Human and Peoples’ Rights on the Rights of Women in Africa (the ‘Maputo protocol’) asserts that access to safe abortion is a human right. Following the Maputo protocol, Ethiopia liberalized its abortion law in 2005. Since then, termination is allowed if it endangers the life or physical health of the pregnant woman, in cases of rape or incest, if she is a minor or mentally or physically disabled, or if the fetus has an ‘incurable and serious deformity’ [1]. Concerning the rape/incest criterion, the law specifically states (art. 552) that ‘the mere statement by the woman is adequate to prove that her pregnancy is the result of rape or incest’.

In Ethiopia, both public and private health institutions offer safe abortion services and healthcare practitioners from different professions are involved in the provision. Private institutions, in which two-thirds of abortions are performed, are often run by non-governmental organizations (NGOs) [2]. Abortion is performed free of charge in public institutions. At the time of data collection, it cost approximately 400–1500 Ethiopian Birr (9–34 US$) in the private institutions. Studies show that Ethiopian abortion services have both expanded and improved in terms of quality since 2005 [3]. While abortion services are at the recommended level in some cities and regions, they are poorly developed rurally and in other regions [4]. In the capital, Addis Ababa, where the abortion rate is the nation’s highest, estimated to be 92 per 1000 women of reproductive age, abortion is performed by institutions ranging from primary health care centers to referral hospitals. Although access to safe abortion has increased since the liberalization of the law, there are still underground unsafe abortion practices. In 2014, an estimated 294,100 induced abortions occurred outside of health care centers in Ethiopia. This is perceived as a significant problem, still contributing to an estimated 10% of maternal mortality [5, 6]. In Sub-Saharan Africa in general, unsafe abortion is still a significant concern and maternal deaths due to unsafe abortions are still high [7].

A recent study with professionals providing abortion in Addis Ababa found that they were familiar with the abortion law but that it gave rise to three main types of moral dilemmas for them: whether abortion should be provided or not; how providers should handle a situation where they suspect that the patient lied in order to qualify for the abortion; and how they should weigh and evaluate different reasons for abortion [8]. The authors state that providers’ assessment of the patient’s reasons for abortion ‘did not always follow the lines of the law’. Many providers were willing to ‘stretch’ the law’s criteria; as one informant put it, ‘the legal part [i.e., the abortion law] has a slightly open door’.

In a national survey mapping moral dilemmas experienced by physicians from Ethiopian public hospitals, abortion was perceived as an important source of dilemmas [9]. In particular, some respondents viewed the abortion law as too restrictive, and it was a dilemma when the woman did not meet the legal criteria for abortion. In another study, health care providers with previous experience with induced abortion were 2.5 times more favorable towards the law than those who were inexperienced [10].

### The present study

The present paper is the third and final paper from an extensive interview study with abortion providers in Addis Ababa. The first paper specifically examined participants’ *views on the moral status of the fetus*, and *the impact of their religious views on their work* [11]. In particular, it was highlighted how the participants attempted to reconcile the tension between the demands of their moral and religious views and their professional obligations. It was found that the providers were left to perform this ‘balancing act’ on their own. Whereas some continued to experience a troubled conscience, others had justified participation in abortion through framing it as helping and preventing harm.

The second paper examined participants’ *views on and experiences with conscientious objection (CO) to abortion* [12]. Despite being forbidden by government regulations, CO was practiced. Supporters of CO claimed that accommodation could often be achieved when colleagues were available to carry out an abortion and related tasks, while opponents saw CO as a potential threat to patients’ access to abortion services.

In the present paper we aim to shed light on experiences of abortion providers with the revised Ethiopian abortion law, including how they view and resolve any moral challenges and dilemmas both related to the law and unrelated. Moral challenges were defined as situations in which there was doubt or disagreement about the right or best course of action. Views on abortion for fetal abnormalities were examined specifically for two reasons. Firstly, experiences from other countries indicate that this gives rise to particular moral challenges; secondly, the topic had not been explored through interviews with providers in Ethiopia before.

## Methods

Methods have been described in detail in the two previous papers [10, 11]. The most salient features of the approach used are summarized here.

### Setting and recruitment

A qualitative design was deemed appropriate for exploring in-depth the experiences and reasoning of healthcare professionals involved in abortion services. The first author recruited practitioners with diverse professional backgrounds working with abortion in a practical and/or administrative capacity in private (NGO) or public healthcare settings in Addis Ababa. Participants worked in one of two public hospitals or seven NGO clinics. The first author approached potential participants by going to their working site, after first getting permission from the local manager. As compensation participants were given 100–200 Ethiopian Birr (equivalent to $2.50–5.00). Informed consent was obtained from each participant.

### Data collection

Interviews were semi-structured, aided by an interview guide which contained open-ended questions on views on abortion, abortion law and abortion for fetal abnormalities, the influence of religion, experienced moral challenges, and conscientious objection. Interviews took place between February and July 2017, at the participants’ workplace, where they were interviewed by the first author in Amharic. Interviews were recorded digitally, transcribed verbatim, then translated into English by an independent Ethiopian researcher fluent in English.

### Analysis

Analysis was performed within the systematic text condensation (STC) framework, a qualitative analysis framework developed by Malterud which builds on DiGiorgi’s method [13]. The method involves four steps:

1. From chaos to themes: the transcripts and field notes were read several times to create an overall impression and identify candidates for main themes.
2. From themes to codes: each unit of meaning was identified and coded according to topic using the nVivo 11 software package. Codes and sub-codes were created.
3. From code to condensation: all units of meaning coded with the same sub-code were then read in order with a view to identifying their meaning and content. This was done by creating so-called ‘artificial quotations’, which are condensed summaries of salient points formulated as if phrased by the participants. All sub-codes were condensed in this way.
4. From condensation to analytic text: the artificial quotations then provided the basis for the final analytic text which was then incorporated into the findings section of the paper. In the analytic text, genuine (not artificial) quotations from the transcripts were used to illustrate and confirm the findings.

The first and fourth authors analyzed the data independently following these steps. They then met to discuss and harmonize their analyses. Three main themes were decided on: ‘differing views on abortion law’, ‘experienced dilemmas’, and ‘abortion justified by fetal abnormalities’.

Data were collected in accordance with conditions in the research ethics approvals and thus also the relevant Norwegian guidelines and regulations. The data were analyzed and findings were reported in accordance with recognized standards for qualitative research, as per the STC framework presented above.

### Research ethics

Ethical approval was obtained. Informed consent was obtained from all participants after providing detailed information on the study both orally and in writing. As the interviews concerned sensitive topics, we limit demographic and other information about individual informants in order to protect their anonymity.

## Results

### Participant background

Thirty healthcare professionals agreed to be interviewed. Of these, most (22) provided abortions directly, while the remaining eight participants had managerial roles in abortion provision, and patient-directed work such as providing contraceptives and post-abortion care. Participants had experience with providing abortion that ranged from two months up to 14 years. The sexes were equally represented with 15 being female and 15 being male. Twenty-one participants identified as Ethiopian Orthodox, five as Protestant/Evangelical, and two as Muslim. One was religiously unaffiliated, and one did not want to disclose his/her views.

### Differing views on the abortion law

A general finding was that there was a range of viewpoints both on the abortion law and on potential moral challenges. Informants’ viewpoints clearly aligned with their moral views on abortion. For instance, informants who regarded abortion as a moral problem also were critical or ambivalent to aspects of the new abortion law thought to be too liberal/open, whereas informants who did not see abortion as a moral problem welcomed liberal interpretations of the law and sometimes found it to be too restrictive.

A majority of participants, especially from private/NGO clinics, stated that they were content with the law, even though they pointed to some shortcomings. They asserted that the most important consequence of the law was that it reduced the incidence of unsafe abortion, and thus saved women’s lives and reduced the number of complications significantly.

Those days abortion was done by nonskilled individuals. … Even if it was by professionals, it was not by skilled professionals. Many of our sisters, mothers have lost their lives. There are some who [have had to] remove their uterus and lost their chance to ever have children. Whose marriage became unstable, who faced psychological problems (#17, female nurse, public hospital).

The good side [of the new law] is that it has helped her to receive a complete treatment by bringing the service to health institutions. It saves many mothers from death (#18, male resident, public hospital).

Other positive effects highlighted were that the law provides freedom of choice to pregnant women, protects patient confidentiality, and reduces delay. It was pointed out as a problem for freedom of choice that many citizens are insufficiently familiar with the law. For several, an important argument in favor of legal access to abortion was that many women with unwanted pregnancies would choose abortion whether it is legal or not, as illustrated in the quote below:

If women have once made up their minds, nothing stops them. Their reason must be respected. That is her right. I have no problem [with that]. Therefore, it is better that we terminate it in a safe way (#20, male resident, public hospital).

Many respondents, while content that the law gives many women access to abortion, would like further liberalization with wider or alternative criteria. Several stated that it was good that they did not have to ask for evidence or witnesses beyond the woman’s statement in relation to the rape criterion.

However, a minority of informants thought that the law went too far in not requiring additional evidence; or in being too liberal, as abortion was still in their view a moral dilemma. They feared that the threshold for seeking abortion had become too low and saw women returning for multiple abortions as a sign of this. They thought that abortion had become de facto accessible on request.

Because we have made it loose, any woman can abort without any check. … What it looks like now is that abortion is legal. It is open. It is not what was intended [when] the law [was passed]. ... Any woman can receive abortion... Even when [it is promoted] the message is that people should not go to private institutions, go to the governmental ones and say that you have been raped (#22, female GYN/OBS resident, public hospital).

The law’s criteria were open to interpretation. This could be seen as an advantage for those who supported liberal access to abortion. For instance, one female health officer from a private/NGO clinic stated:

It can be said that [the health criterion] indirectly has allowed everything. … [Abortion] is not permitted completely, but it is permitted indirectly. For example, if you say mentally, it means that it is allowed if it involves stress. The majority of pregnancies are stressful. They come because they are stressed. When you think like that, [abortion] is allowed (#6, female health officer, private clinic).

### Experienced dilemmas

Participants were asked about which moral challenges, if any, they encountered in their work with abortion. Although most had experienced several challengeses, some stated that they had not encountered any significant moral challenges. A few pointed out that the decision whether to choose and perform abortion by its nature is a moral issue: “Abortion is an ethical dilemma both [for the patient] and the one who performs it” (#21, male GYN/OBS, public hospital).

The major moral dilemmas typically involved the interpretation and application of the law’s criteria for abortion. Some admitted that they interpreted the criteria widely. Others appeared to feel burdened by expectations and pressure from patients in cases where criteria were not met, or where they were uncertain whether criteria were met. Sometimes this led to discussions and disagreement among colleagues. In general, participants from public hospitals appeared somewhat less liberal and less comfortable with applying a broad (inclusive) interpretation of the law’s criteria than did participants from the private/NGO sector.

Sometimes they were expected to perform abortions beyond the law’s gestational stage of 28 weeks:

We come across problems quite often, especially, a woman admitted late in the pregnancy in the name of safe abortion. … This is not legal. … I cannot assist in a delivery of [a] 1 kg [child] and call it an abortion. We have had a lot of conflicts over this issue. We know it. Things that are not acceptable for your conscience are done (#22, female GYN/OBS resident, public hospital).

In some cases, the law’s criteria for abortion were not clearly met. A few of the participants would then reject performing abortion, whereas others would sometimes accept it.

If she comes for abortion with no reason, I do not do it because I do not accept it … but I transfer it to one who does it. Because I do not believe that is her right. Many of us do not do it (#22, female GYN/OBS resident, public hospital).

To be honest, if she says that she does not want to give birth, … we do not [turn her away]. We perform the abortion. (#1, female nurse, private clinic)

There are [criteria] stated in the law. There are also some who approach us because of other factors. Many times, we do not base our service on the law. We base it on the case which the woman who approaches us tells us. We do not assess whether Ethiopian law allows that or not (#4, female nurse, private clinic).

As noted above, some participants remarked that in the case of rape the law does not require further evidence than the woman’s own word that the pregnancy was due to rape. They expressed that this could potentially give women seeking abortion incentives to lie in order to fulfill the law’s criterion. Similarly, a few participants claimed that patients sometimes lied about their age, claiming to be minors when they clearly were not, in order to comply with the law’s age criterion. This led to dilemmas for practitioners.

The bad side [of the law] is that it makes it liberal. If a woman lies intentionally because the law is on her side, she is given the service. That affects us a bit. I have seen some who attempt suicide when they are told it is too late. If she is 40 but claims to be 13, I am obliged to carry it out, even if I know that she is not telling the truth. It opens up for things. Which means any woman as long as she knows where the service is offered, she can get it. I think that makes [the law] a bit liberal. It affects us. Other than that, the good side [i.e., the positive aspects of the law] weighs more (#18, female GYN-OBS resident, public hospital).

Whereas most moral dilemmas experienced were directly related to interpretation and application of the abortion law, some dilemmas were not. Specifically, dilemmas arose when professionals became involved in a patient’s quarrels with partner or family members. Some patients were pressured to abort or to continue the pregnancy against their preference. Furthermore, many pointed to the low level of awareness of family planning and contraception in the population as a moral problem.

### Abortion justified by fetal abnormalities

When asked about termination of pregnancy in cases of fetal abnormalities, the majority of the participants said that they believed termination should be performed/offered. They highlighted serious negative consequences of having children with abnormalities for the woman, her family, and also society. A few explicitly pointed to the economic burdens for society, and several cited the shortcomings of the Ethiopian healthcare system which would make it difficult to give the child proper care.

If there is disability, it has to be terminated. If it is early, the mother can also be affected psychologically. It would be difficult. [If] it is early, it is better to terminate quickly. Even [some] mothers who deliver a baby with cleft lip do not want to have another child (#10, female nurse, public hospital).

Had the health system of our country been good, [the child] could grow up if delivered. But we do not have [a good health system]. If they are delivered the problem comes to the family, to the society, to the country (#11, male public health specialist, private clinic).

There were only a few who expressed serious ambivalence about termination in case of fetal abnormality:

I want the decision to be taken based on the family situation and economic ability. However, this collides with the rights of the disabled. When you see it from a different angle there needs to be a balance. It needs to be approached from the human right aspect. It is very problematic (#6, female nurse, private clinic).

Many were clear that one should distinguish between lethal malformations and milder abnormalities. Termination was considered the right choice for the former, whereas views differed on the latter. Informants were invited to reflect on Down syndrome as a specific case. Most favored termination, whereas a minority did not or were ambivalent.

I believe that Down syndrome has to be aborted. It is [costly] for the country. … I think it is reasonable to abort that child (#15, male, public health specialist, private clinic).

The degree [of being affected by Down syndrome] determines it. If it is severe, it is better that it is not born. But those who are mild or moderate, it is preferred that they live [and receive] training and support. They [can be], to a degree, independent (#9, female nurse, public hospital).

Participants were unanimous in wanting to leave the decision whether to terminate a pregnancy with abnormalities to the woman herself as illustrated in this remark:

It is the mother who takes care of [the child] at the end of the day. ... It means that a decision is made on her. Therefore, in my view, she should have a say (#21, male GYN/OBS, public hospital).

## Discussion

Exploring the ways in which the revised Ethiopian abortion law was being interpreted and experienced among abortion providers shed some light into the nuances of their practices and the resolution of moral challenges and dilemmas [14]. Most of the moral challenges experienced by the professionals turned out to spring from the interpretation and application of the abortion law, consistent with previous research [8, 9].

### Interpreting the abortion law’s criteria

The law’s criteria are brief and open to interpretation which has been viewed as problematic in how criteria are applied [15].﻿ For instance, even though the law does not allow termination of pregnancy due to socioeconomic reasons, this might be taken to be *indirectly* allowed through the criterion of the mother’s health, since all pregnancies represent a certain risk to the woman’s health. In a study from the Guraghe zone in Southern Ethiopia, 36.7% of women having undergone abortion claimed that the choice of abortion was due to economic reasons [16].

As a result of the law being broadly open for interpretation, abortion is viewed as de facto available on request, and from a providers’ perspective somewhat burdensome because potentially the clients choose where to draw the line. Both of these findings are in line with McLean’s study [8].

As Blystad and colleagues also argue, the interplay between law, health policy and implementation is complex, and in the case of Ethiopian abortion regulation this has led to significant room for individual providers’ discretion [17]. Because some respondents acknowledged practicing ‘abortion on request’ with little regard for whether the law’s criteria were met, it appears that it is the individual practitioner’s stance that could potentially determine whether abortion will be provided or not. This stance might again be influenced by the institution’s policy and the practitioner’s moral values including moral views on abortion.

If the authorities want to avoid such consequences and restrict the space for each practitioner’s interpretation, they could consider making the guidance more precise and less ambiguous, either through amending the law or through providing more detailed guidance in how it should be interpreted. However, this might not succeed if individual providers claim the authority to determine whether an abortion is justified without deference to the law’s criteria.

### Abortion in light of fetal abnormalities

None of the participants mentioned abortion for fetal abnormalities as a moral challenge they experienced prior to being prompted to reflect on the issue. They referred to the moral value of the fetus and the rights of the disabled versus values of the woman’s autonomy and the difficulty in providing the child with requirements for a good life. However, abortion was considered a reasonable alternative in the case of fetal abnormality (albeit different attitudes concerning the severity of the disability), and they all appeared to want to leave the choice to the woman. Their main arguments align with Muderedzi and Ingstad’s observations that “[d]isability can cause poverty by preventing the full participation of disabled people in the economic and social life of their communities, especially if the proper supports and accommodations are not available” [18]. Seen this way, improvements in welfare, health and social care might make it more feasible for Ethiopians to choose to complete the pregnancy in case of fetal abnormality. Such improvements would also improve women’s autonomy, as they would enable a choice between different options that are actually realistic.

In a study of the preferences of pregnant women at an Addis Ababa hospital, 89% would want prenatal testing, and more than 60% of the women reported interest in termination in case of anencephaly, lethal conditions, severe intellectual disability, hemoglobinopathy, and amelia [19]. Conversely, in a 2013 nationally representative study from South Africa, more than half of the respondents (55%) considered it always wrong to choose abortion for fetal abnormalities [20]. The attitudes of the Ethiopian population, including health professionals on these issues should also be assessed in surveys.

### Strengths and limitations

The qualitative design provided in-depth accounts of moral challenges and dilemmas experienced by providers practicing induced abortion. Only providers in Addis Ababa have been interviewed; experiences in other parts of Ethiopia, including rural settings could be different. Exploring actual practices directly fell out the scope of the current study, and thus we couldn’t verify or triangulate participants’ own accounts. It is likely that patients seeking abortion could be evaluated differently depending on to which institution they go and which practitioner they meet.

## Conclusion

Although most abortion practitioners in our study considered the 2005 abortion law a clear improvement, they acknowledge new dilemmas. The large space for abortion participants’ individual interpretation and application of the abortion law’s criteria appears to place a considerable authority, burden and responsibility on each practitioner. For patients, it might conceivably mean that access to abortion is dependent on the views and practices of the practitioner encountered.

## Data Availability

The data generated during and analyzed during the current study are not publicly available as individual informants might be identified from the interview transcripts. Any requests about availability of the data should be directed to the corresponding author.

## Abbreviations

CO: Conscientious objection
NGO: Non-governmental organisation
STC: Systematic text condensation
